# Mono-(2-ethylhexyl) Phthalate (MEHP)-Induced Telomere Structure and Function Disorder Mediates Cell Cycle Dysregulation and Apoptosis via c-Myc and Its Upstream Transcription Factors in a Mouse Spermatogonia-Derived (GC-1) Cell Line

**DOI:** 10.3390/toxics11050448

**Published:** 2023-05-10

**Authors:** Fangji Zhou, Chengwei Guo, Lingqiao Wang, Guowei Zhang, Jia Wang, Weiyan Chen, Ke Cui, Yao Tan, Ziyuan Zhou

**Affiliations:** Department of Environmental Health, College of Preventive Medicine, Army Medical University (Third Military Medical University), Chongqing 400038, China

**Keywords:** mono-(2-ethylhexyl) phthalate, telomere dysfunction, G_0_/G_1_ phase cell cycle arrest, apoptosis, TERT, c-Myc, male reproductive toxicity

## Abstract

As a typical environmental endocrine disrupting chemical (EDC), di-(2-ethylhexyl) phthalate (DEHP) is thought to be related to reproductive disorders, especially in males. Growing evidence suggests that various EDCs may result in an impaired telomere structure and function, which is associated with male infertility. However, the adverse effect of DEHP on telomeres in male reproductive cells has rarely been studied, and the related mechanisms remain unclear. In this study, we tested the effects of mono-(2-ethylhexyl) phthalate (MEHP), the primary metabolite of DEHP, on telomere dysfunction in mouse spermatogonia-derived cells (GC-1) and the potential role of TERT and c-Myc in MEHP-induced spermatogenic cell damage. Results showed that MEHP induced cell viability inhibition, G_0_/G_1_ phase cell cycle arrest, and apoptosis in GC-1 cells in a dose-dependent manner. Shortened telomeres, reduced telomerase activity, and decreased expression of TERT, c-Myc, and upstream transcription factors of c-Myc were also observed in the MEHP-treated cells. In conclusion, it can be concluded that TERT-mediated telomere dysfunction may contribute to MEHP-induced G_0_/G_1_ phase cell cycle arrest and apoptosis in GC-1 cells through the impairment of c-Myc and its upstream transcription factors.

## 1. Introduction

Di-(2-ethylhexyl) phthalate (DEHP), as one of the most widely studied phthalate derivatives, is frequently used as a plasticizer in polyethylene (PE) plastics and polyvinyl chloride (PVC) plastics [1]. The non-covalent binding of Phthalic acid esters (PAEs) to plastic molecules makes them susceptible to escaping from plastic materials under certain conditions and are constantly being released into the surrounding environment, polluting the air, water, and soil [2]. Therefore, humans are inevitably exposed to DEHP through breathing, dietary intake, and dermal absorption [3]. Accumulating evidence indicates that PAEs have a definite reproductive toxic effect. Specifically, epidemiological studies have shown that exposure to DEHP is associated with a decrease in semen quality and a reduction in serum testosterone levels [4,5]. Previous studies in animal models have clearly demonstrated that direct exposure to DEHP has a toxic effect on the male reproductive system, resulting in testicular atrophy, decreased sperm count, and reduced sperm viability [6,7]. However, less is known about the genotoxic effect on the male reproductive system caused by DEHP.

Generally, DNA damage caused by environmental or endogenous genotoxic agents represents a serious survival challenge for cells [8]. DEHP and its metabolites have been well-documented to directly provoke oxidative stress, disrupt the DNA integrity of sperm, induce DNA damage, and result in reproductive toxicity [9,10]. Telomeric DNA is composed of a repetitive sequence 5′-TTAGGG-3′, enriched in guanine base G, which makes telomeres more vulnerable to breakage by reactive oxygen species attack. Keeping the telomere length at a certain level is an imperative prerequisite for cells to be able to continuously divide. During DNA replication in somatic cells, telomere loss occurs in each round of cell division due to the inability of DNA polymerase to replicate chromosome ends and prevent cell proliferation infinitely by inducing differentiation, cycle arrest, replicative senescence, or apoptosis [11,12]. To avoid telomere loss, telomerase is indispensable. Telomerase is a ribonucleoprotein that includes the telomerase reverse transcriptase (TERT) and the telomerase RNA (TERC). Suppressing TERT markedly diminishes telomerase activity, shortens the telomere length, and increases apoptosis. Conversely, activating TERT expression obviously improves telomerase activity and promotes telomere elongation and cell proliferation. Together, these findings indicate that TERT is a rate-limiting factor for telomerase activity and performs a critical role in maintaining telomerase function [13,14]. There are multiple transcription factor binding sites in the promoter region of the TERT gene including c-Myc, Mad1, SP1, and estrogen response elements. c-Myc is one of the essential transcription factors, which has been demonstrated to be involved in cell proliferation and growth as well as in the processes of differentiation and apoptosis. Chromatin immunoprecipitation shows that c-Myc and Max-formed heterodimers can directly interact with the hTERT promoter [15]. The estrogen/estrogen receptor, another transcription factor on the TERT gene promoter, can also indirectly activate TERT by upregulating the expression of c-Myc [16]. The ability of c-Myc to activate hTERT gene expression and telomerase activity leads to c-Myc-dependent cell immortalization. These studies indicate that TERT is a direct target of the c-Myc protein.

The stability of the telomere structure and function contributes to protect germ cells from growth inhibition, senescence, apoptosis, and even death. Telomerase and TERT are highly expressed in germ cells, especially in spermatogonia [17]. It is well-documented that telomeres are very sensitive to the external environment and that various environmental and occupational exposures related to air pollution [18], persistent organic pollutants [19], endocrine disruptors [20], and heavy metal contaminants [21] can disrupt telomere dynamic homeostasis and induce telomere length shortening. Thus, in conjunction with the literature, an important conclusion can be drawn—telomeres may be a significant target for genetic damage induced by various environmental pollutants. However, research on telomere damage caused by environmental pollutants is limited not only to the respiratory system and peripheral blood lymphocytes, but also to the male reproductive system. Telomere homeostasis is essential for the formation of spermatozoa [22]. Telomerase are sensitive to environmental influences. Environmental pollutants such as phthalates [1], polycyclic aromatic hydrocarbons (PAHs) [23], brominated flame retardants [24], PM2.5 [25], and fluoride [26] have been clearly proven to induce telomere damage in the male reproductive system, which may be associated with abnormalities in telomere structure and function such as shortened telomere length and reduced telomerase activity in spermatogenic cells. However, only a few papers have reported that PAEs can cause telomere disruption in spermatogenic cells. These studies stay at the effect level and do not delve into the essential reasons for telomere damage caused by these pollutants, so the underlying molecular mechanisms are unclear and still need to be further explored.

Therefore, based on the fact that telomerase activity and TERT are highly expressed in spermatogonia, we chose GC-1 mouse spermatogonia as the subject of our study. This experimental design will focus on the mechanisms associated with telomere damage in male reproductive damage caused by PAEs. By establishing in vitro cellular assays to explore the impacts of MEHP on germ cell toxic effects and telomere injury and delve into the mechanisms underlying this damaging influence caused by MEHP, this study will provide more evidence for the prevention of male reproductive toxicity induced by the compounds of PAEs.

## 2. Materials and Methods

### 2.1. Chemicals and Antibodies

MEHP was purchased from Sigma Aldrich (Saint Louis, MO, USA, CAS: 4376-20-9, 97% pure). Commercially available antibodies against CDK4 (sc-56277) and CYCLIN D1 (sc-8396) were obtained from Santa Cruz Biotechnology (Santa Cruz, CA, USA). The c-Myc (#18583) and Actin (#8457S) antibodies were purchased from Cell Signaling Technology (Boston, MA, USA). The anti-Max (ab199489) antibody was obtained from Abcam (Cambridge, MA, USA). The anti-TERT (bs-0223R), Bax (bsm-52316R), and Bcl-2 (bsm-52833R) antibodies were obtained from Bioss (Beijing, China). The CTCF (A19588), STAT3 (A19566), and ESR1 (A12976) antibodies were from ABclonal Technology (Wuhan, China). The anti-C-JUN (ET1608-3) and FOXA1 (ET1702-89) antibodies were from HuaAn Biotchnology Co., Ltd. (Hangzhou, China). Horseradish peroxidase (HRP)-conjugated goat anti-rabbit IgG (A0208) and HRP-conjugated goat anti-mouse IgG (A0216) were from Beyotime Institute of Biotechnology (Shanghai, China).

### 2.2. Cell Culture and Treatment

The mouse spermatogonia-derived GC-1 spd (ts) cells (GC-1 cells) were purchased from the American Type Culture Collection (ATCC, Rockville, MD, USA). The cells were cultured in DMEM medium (HyClone, Logan, UT, USA) with 10% FBS and 1% penicillin-streptomycin at 37 °C in a humidified atmosphere containing 5% CO_2_. Cells were treated with MEHP (50, 100, 200, and 400 μM) dissolved in DMSO. Control cells received DMSO (0.05% final concentration) alone. GC-1 cells presented a semi-adherent and semi-suspended state under natural growth conditions. Each cell culture experiment was repeated at least three times.

### 2.3. Cell Viability

The cytotoxicity of MEHP to GC-1 cells was assessed using the Cell Counting Kit-8 (CCK-8; DOJINDO, Kumamoto, Japan) assay. According to the manufacturer’s recommendations, the cells were seeded into a 96-well plate at a density of 10,000 cells per well for 24 h and then treated with different concentrations of MEHP for 48 h. The cells were then incubated with CCK-8 solution for another 2 h. The cell viability was expressed as the optical density detected at 450 nm using an enzyme labeling instrument.

### 2.4. Cell Cycle Assay

Cell cycle was evaluated using the cell cycle and apoptosis kit (Beyotime, China) as described by the manufacturer’s instructions. After MEHP exposure, cells were collected, washed, and then fixed overnight in ice-cold 70% ethanol at 4 °C. Fixed cells were washed with cold PBS, incubated with 0.5 mL propidium iodide staining solution at 37 °C for 30 min, and the cell distribution was then detected and analyzed by flow cytometry (AccuriC6, BD Biosciences, San Jose, CA, USA) in 2 h.

### 2.5. Apoptosis Assay

Apoptosis was evaluated using the Annexin V-FITC Apoptosis Detection Kit from BD Pharmingen (San Jose, CA, USA) following the manufacturer’s instructions. After MEHP treatment, cells were harvested and incubated with Annexin V-FITC and propidium iodide (PI) for 15 min at room temperature in the dark. Subsequently, the Annexin V-positive cells were analyzed by flow cytometry in 30 min.

### 2.6. Telomere Length Measurement

Genomic DNA was isolated from cells using a DNA/RNA Isolation Kit (Omega Bio-Tek Inc., Norcross, GA, USA) according to the manufacturer’s instructions. The relative telomere length of cells was then measured by determining the ratio of the telomere repeat copy number to the single copy gene copy number (T/S ratio) using real-time quantitative PCR (RT-qPCR). RT-qPCR was performed with the SYBR Master Mix in a final reaction volume of 20 μL containing 20 ng of genomic DNA. The primer sequences are shown in the Supplementary Materials (Table S1).

### 2.7. Real-Time Quantitative PCR (RT-qPCR)

RNA extraction was conducted according to the manufacturer’s protocol. The Total DNA/RNA Isolation Kit (Omega Bio-Tek Inc., Norcross, GA, USA) was used to extract RNA and the iScript cDNA Synthesis Kit (BIO-RAD, USA) was used to reverse transcribe the RNA to cDNA followed by real-time quantitative PCR using the SYBR Master Mix. The final reaction volume was 20 μL, and the relative expression of genes was analyzed by the 2^−∆∆CT^ method. *Beta-actin* (*β-actin*) was used as the housekeeper gene. The primer sequences are shown in the Supplementary Materials (Table S2).

### 2.8. Western Blot Analysis

After treatment, the cells were harvested and lysed in IP lysis buffer (Beyotime, China) on ice for 30 min. The protein concentration was determined by the BCA assay (Beyotime, China), and 30–40 μg of protein was separated by 12% SDS-PAGE and transferred to a PVDF membrane (Millipore, Bedford, MA, USA). The membranes were then soaked in blocking buffer (5% skim milk) at room temperature for 2 h and incubated with primary antibodies overnight at 4 °C. After three washes, the membranes were incubated with HRP-conjugated secondary antibodies for 1.5 h at room temperature. The membranes were then washed three times again, and the signal was detected using an enhanced chemiluminescence (ECL) detection kit.

### 2.9. Telomerase Activity Measurement

The telomerase activity of the GC-1 cells was determined using the mouse telomerase (TE) ELISA kit (meibiao, Jiangsu, China). GC-1 cells were collected and resuspended with PBS. The supernatant was obtained by centrifugation after repeated freeze–thaw. Then, the supernatant as well as a series of dilutions from the standard substances were added to the wells coated with mouse TE-specific antibodies. The HRP conjugate reagent was added to each well and incubated at 37 °C for 60 min followed by five washes. Chromogenic agents A and B (50 μL) were added to each well and incubated at 37 °C for 15 min away from light. The reaction was terminated by a stop solution, and the OD value was measured at 450 nm.

### 2.10. Statistical Analysis

All experiments were repeated at least three times, and the resulting data were expressed as the mean ± standard deviation (SD). SPSS 25.0 software was used for the data analysis. One-way analysis of variance (ANOVA) was used to compare the differences between the experimental and control groups. A value of *p* < 0.05 indicated that the variance was statistically significant.

## 3. Results

### 3.1. MEHP Reduces Cell Viability and Induces G_0_/G_1_ Phase Cell Cycle Arrest and Apoptosis in GC-1 Cells

**Figure 1:**
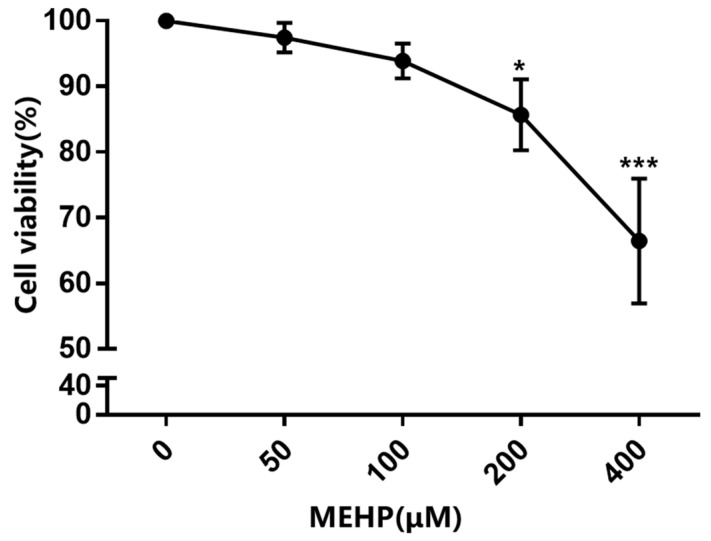
MEHP reduces the cell viability in GC-1 cells. GC-1 cells were treated with various concentrations of MEHP (0, 50, 100, 200, and 400 μM) for 48 h. Cell viability was assessed using a Cell Counting Kit-8 (CCK-8) assay and expressed as the percentage of the optical density of MEHP-treated cells compared with that of the control cells (100% viability). Results are expressed as mean ± SD, n = 3. * *p* < 0.05, *** *p* < 0.001 versus the control group treated with DMSO.

**Figure 2:**
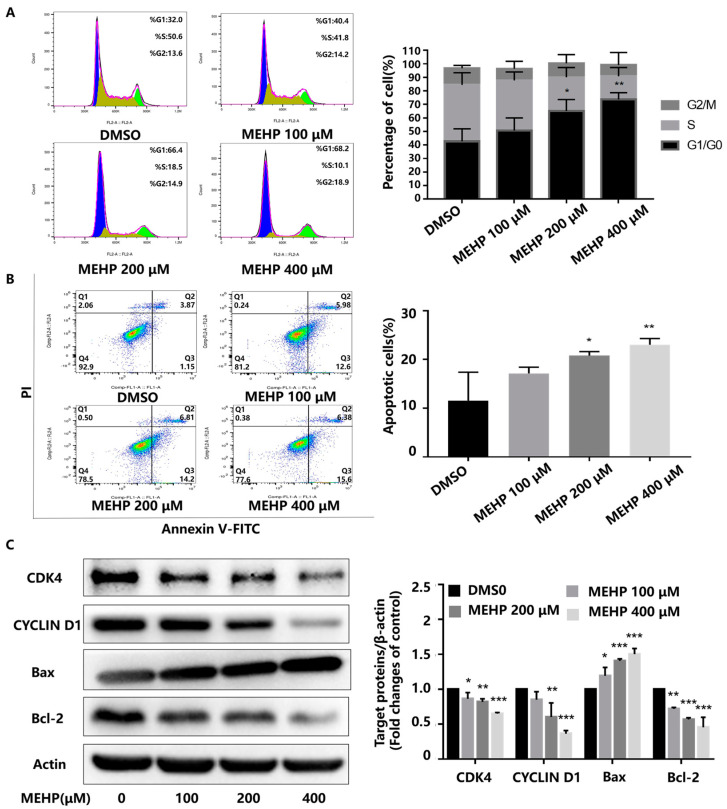
MEHP induces G_0_/G_1_ phase cell cycle arrest and apoptosis in GC-1 cells. (**A**) GC-1 cells treated with various concentrations of MEHP for 48 h were subjected to cellular cycle analysis using flow cytometry with PI staining. Representative flow cytometry plots are presented to the left of panel (**A**). The percentage of cell cycle is shown in the bar graph to the right of panel (**A**). (**B**) Apoptotic cells were detected by Annexin V-FITC/PI staining combined with flow cytometry analysis. Representative flow cytometry scatter plots are shown to the left of panel (**B**), and the percentage of apoptotic cells (Annexin V-positive cells) is shown in the bar graph to the right of panel (**B**). (**C**) The expression levels of the target proteins CDK4 and CYCLIN D1 that regulate the cell cycle from the G_1_ phase to S phase and apoptosis-related proteins Bax and Bcl-2 were examined using Western blot and quantified relative to β-actin by densitometric analysis of the band. Results are expressed as the mean ± SD, n = 3. * *p* < 0.05, ** *p* < 0.01, *** *p* < 0.001 versus the control group treated with DMSO.

In the present study, the survival of GC-1 cells after treatment with varying concentrations of MEHP (50, 100, 200, and 400 μM) for 48 h was detected by the CCK-8 assay. As shown in Figure 1, MEHP reduced the cell viability in a dose-dependent manner when the concentration was higher than 100 μM compared with the DMSO-treated control. The cell viability of the 100 μM MEHP group decreased to about 94%, while those of the 200 and 400 μM MEHP groups were reduced to about 86% and 67%, respectively (*p* < 0.05). To clarify whether the MEHP-induced decrease in the cell viability of GC-1 cells was related to cell cycle arrest or apoptosis, we examined the cell cycle and apoptosis using PI fluorescent staining or Annexin V-FITC combined with flow cytometry, respectively. Our study revealed that MEHP treatment for 48 h induced G_0_/G_1_ phase cell cycle arrest in GC-1 cells and that the percentage of cell cycle arrest was positively correlated with the MEHP concentration, with significant effects in the 200 and 400 μM groups (Figure 2A). Moreover, MEHP treatment also caused cellular apoptosis in a dose-dependent manner following 48 h of exposure (Figure 2B). The results of the Western blot analysis further supported the results of the flow cytometry. The levels of two important regulatory proteins (cyclin-dependent kinase 4 [CDK4] and CYCLIN D1) that are necessary for the transformation of the G_0_/G_1_ phase to S phase obviously decreased. Meanwhile, after MEHP exposure, the expression level of Bax, a key protein that promotes apoptosis, markedly increased, while the expression level of anti-apoptotic protein Bcl-2 was significantly reduced (Figure 2C). Similarly, as shown in Figure S1, the mRNA expression levels of *Cdk4*, *Cdk6*, *Ccnd1*, and *Rb1* declined with the increase of MEHP. Together, our results demonstrate that MEHP resulted in a decrease in cell viability and an increase in G_0_/G_1_ phase cell cycle arrest and apoptosis in the GC-1 cells.

### 3.2. MEHP Induces Telomere Structure and Function Disorder in GC-1 Cells

**Figure 3:**
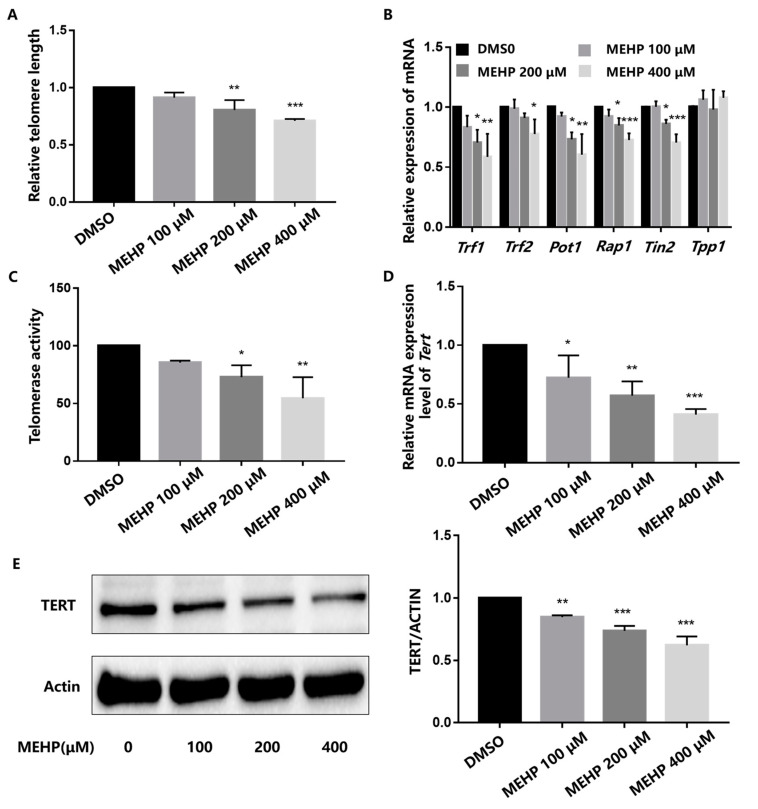
MEHP damages the telomere structure and results in telomere dysfunction in GC-1 cells. (**A**) GC-1 cells were exposed to different concentrations of MEHP for 48 h. Genomic DNA were then extracted from the cells and the relative telomere length (T/S ratio) was assessed by real-time quantitative PCR (RT-qPCR). (**B**) Changes in the mRNA expression levels of *Trf1*, *Trf2*, *Pot1*, *Rap1*, *Tin2*, and *Tpp1* in GC-1 cells were measured by RT-qPCR after 48 h of MEHP treatment. *Beta-actin* (*β-actin*) was used as the housekeeper gene. (**C**) Telomerase activity was obtained by ELISA in the GC-1 cells after MEHP treatment. (**D**) GC-1 cells were treated with different concentrations of MEHP for 48 h, and the mRNA expression of *Tert* was evaluated by RT-qPCR. *Beta-actin* (*β-actin*) was used as the housekeeper gene. (**E**) After exposure to MEHP at various concentrations for 48 h, the protein expression level of TERT was detected by Western blot and quantified relative to β-actin by densitometric analysis of the band. The results are presented as the mean ± SD, n = 3. * *p* < 0.05, ** *p* < 0.01, *** *p* < 0.001 versus the control group treated with DMSO.

Because cell growth arrest and apoptosis have been proven to be mediated by the induction of telomere dysfunction (i.e., short telomeres), we analyzed the effect of MEHP on the telomere length in male germ cells after 48 h of exposure at different concentrations. The relative telomere length was assessed using RT-qPCR by determining the T/S ratio. Specifically, telomeres were significantly shortened in cells treated with 200 or 400 μM of MEHP. However, although the telomere length was shortened in the 100 μM dose group, the variation was not statistically significant (Figure 3A). We further detected the changes in the telomere-related multiprotein complex (shelterin). The results showed that the mRNA expression levels of shelterin including *Trf1*, *Trf2*, *Pot1*, *Rap1*, and *Tin2* decreased, especially in the 400 μM dose group, but there was no appreciable change in the *Tpp1* mRNA expression in all dose groups (Figure 3B). To explore the effect of MEHP on the telomerase activity, the GC-1 cells were subjected to telomerase (TE) ELISA analysis after treatment with MEHP. The results demonstrate that telomerase activity was obviously reduced in the 200 and 400 μM exposure groups (Figure 3C). The telomerase reverse transcriptase (TERT) is a pivotal determinant of telomerase activity and telomere length maintenance. RT-qPCR analysis showed that the mRNA expression of *Tert* was downregulated in a dose-dependent manner after 48 h of MEHP treatment (Figure 3D). Furthermore, MEHP also lowered the protein level of TERT in all dose groups (Figure 3E). Collectively, these data suggest that MEHP can cause telomere dysfunction in GC-1 cells, and lead to the inhibition of telomerase activity and decrease in TERT expression.

### 3.3. MEHP Inhibits c-Myc Expression in GC-1 Cells

**Figure 4:**
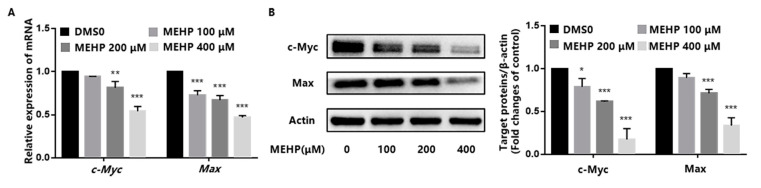
MEHP decreases the expression of c-Myc in GC-1 cells. (**A**) After 48 h of MEHP treatment, the mRNA expression levels of *c-Myc* and *Max* were detected by real-time quantitative PCR. *Beta-actin* (*β-actin*) was used as the housekeeper gene. (**B**) GC-1 cells were exposed to different concentrations of MEHP for 48 h, and the protein expression levels of c-Myc and Max were detected by Western blot and quantified relative to β-actin by densitometric analysis of the bands. The results are expressed as the mean ± SD, n = 3. * *p* < 0.05, ** *p* < 0.01, *** *p* < 0.001 versus the control group treated with DMSO.

c-Myc is a proto-oncogene that promotes cell proliferation and growth as well as a positive regulator of telomerase activity [27,28]. Specifically, the c-Myc protein can directly activate TERT through a protein dimer formed by binding to the Max protein [15]. Estrogen, another positive regulator of telomerase activity, can also indirectly promote TERT expression by activating the expression of c-Myc [16]. These results suggest that TERT is a direct target of c-Myc. As shown in Figure 4A, the mRNA expression levels of *c-Myc* and *Max* were reduced in a dose-dependent manner after the GC-1 cells were exposed to various concentrations of MEHP for 48 h. Simultaneously, the protein expression levels of c-Myc and Max also decreased with the increasing concentrations of MEHP (Figure 4B).

### 3.4. Effect of MEHP Exposure on c-Myc Upstream Transcription Factors in GC-1 Cells

**Figure 5:**
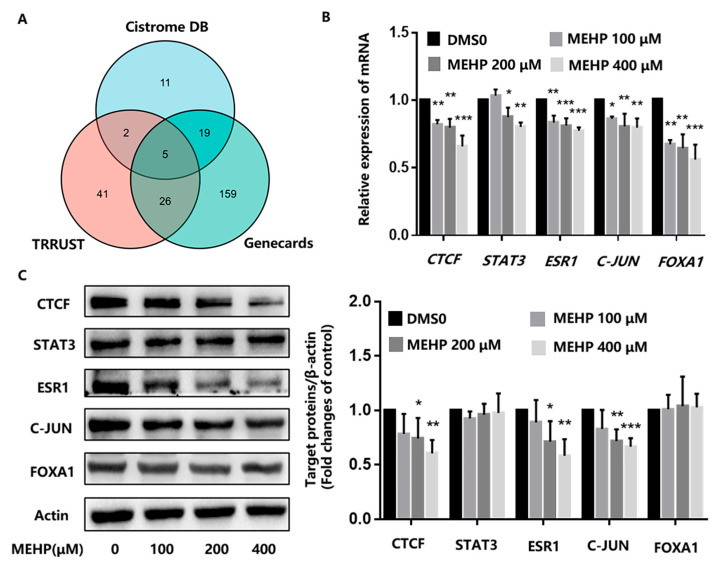
MEHP inhibits c-Myc transcriptional regulators in GC-1 cells. (**A**) The Cistrome DB, TRRUST, and Genecards databases were screened for c-Myc upstream transcription factors and the results of the unique and common parts between each group were visualized using Venn diagrams. (**B**) GC-1 cells were exposed to different concentrations of MEHP for 48 h, and the mRNA expression levels of *Ctcf*, *Stat3*, *Esr1*, *C-jun*, and *Foxa1* were detected by RT-qPCR. *Beta-actin* (*β-actin*) was used as the housekeeper gene. (**C**) After 48 h of MEHP treatment, the relative protein levels of CTCF, STAT3, ESR1, C-JUN, and FOXA1 in the GC-1 cells were detected by Western blot, and the expression levels of these target proteins relative to β-actin were quantified by densitometric analysis of the bands. The results are expressed as the mean ± SD, n ≥ 3. * *p* < 0.05, ** *p* < 0.01, *** *p* < 0.001 versus the control group treated with DMSO.

The transcription factors of c-Myc were queried in the Cistrome database (http://cistrome.org/db, accessed on 13 October 2022), TRRUST database (transcriptional regulatory relationships unraveled by sentence-based text mining, http://www.grnpedia.org/trrust, accessed on 13 October 2022), and the Genecards database (https://www.genecards.org/, accessed on 13 October 2022). The Cistrome database is a resource containing human and mouse cis-regulatory information derived from DNase-Seq, ChIP-Seq, and ATAC-Seq chromatin profiling assays to obtain the information on gene regulatory analysis. The TRRUST database currently contains 8015 interactions between 748 TF genes and 1975 non-TF genes. The *p* values were calculated with the hypergeometric test. A *p* value < 0.05 was deemed as a statistically significant difference. Afterward, we performed the R package of “ggplot2 [3.3.6] and VennDiagram [1.7.3]” to visualize the results of the unique and common parts between each group. As shown in the Venn diagram in Figure 5A, we found that five transcription factors, namely, CTCF, STAT3, ESR1, C-JUN, and FOXA1, could be detected in the three databases above-mentioned. First, we assessed the mRNA levels of these five genes by RT-qPCR after the GC-1 cells were exposed to different concentrations of MEHP for 48 h. The outcomes revealed that MEHP treatment reduced the mRNA expression of *Ctcf*, *Stat3*, *Esr1*, *C-jun*, and *Foxa1* in a dose-dependent manner (Figure 5B). In addition, the Western blot results showed that the protein expression levels of CTCF, ESR1, and C-JUN decreased with an increase in the concentrations of MEHP, especially in the 200 μM and 400 μM dose groups (Figure 5C). Collectively, these data indicate that the MEHP-induced reduction of c-Myc may be related to the damage to upstream transcriptional regulators of c-Myc.

## 4. Discussion

Male reproduction is a complex process influenced by chemical and socio-psycho-behavioral factors that act through different mechanisms. DEHP is a common environmental endocrine disruptor that widely exists in our daily lives. Humans can access DEHP through the gastrointestinal tract, lungs, and skin, with the gastrointestinal tract as the primary route of absorption. After oral ingestion, a small proportion of DEHP is absorbed directly in its original form and the majority is hydrolyzed to the mono-ester metabolite MEHP in the gastrointestinal tract by pancreatic enzymes and intestinal lipases. A portion of MEHP is absorbed directly into the blood through the intestine and then distributed via the blood to the liver, kidneys, fat, testes, and other tissues. The other part of MEHP is metabolized in the liver by cytochrome P450 or the UDP-glucuronosyltransferase (UGT) enzyme to the secondary metabolites 2CX-MMHP, 5CX-MEPP, 5OH-MEHP, and 5OXO-MEHP [29,30]. Approximately 67% of DEHP in the body is excreted in the urine as MEHP, and thus MEHP can be used as a biomarker of DEHP exposure levels. Both DEHP and MEHP have toxic effects, and the toxicity potency of MEHP is more than 10 times that of DEHP. In testicular tissue, MEHP, the primary metabolite of DEHP, cannot be further metabolized, accumulates significantly in testicular tissue, and exerts reproductive toxicity. In recent years, a large number of studies have shown that DEHP has toxic effects on the male reproductive system. Specifically, DEHP induces significant changes in testicular histomorphology, decreases the testicular organ coefficient and sperm count, increases testicular cell apoptosis, and enhances the oxidative stress level of testicular tissue [9,31,32]. A study by Zhu et al. showed that DEHP can cause structural disruption of testicular tissue, the shedding of germ cells, and the reduction in sperm cell numbers in male mice, which is consistent with the conclusions of previous studies [1]. Although the damaging effects of DEHP on the male reproductive system have been confirmed, the molecular events of spermatogenic cell damage and the underlying molecular mechanisms remain unclear and need to be elucidated. Therefore, in this study, we used MEHP, the active metabolite of DEHP, to explore its direct effects on germ cells.

Telomeric DNA is a non-coding sequence rich in guanine G and is vulnerable to damage by reactive oxygen species, making it more susceptible to internal and external factors [33]. Available epidemiological evidence suggests that both DEHP and MEHP, two environmental endocrine disruptors, are associated with changes in the cell telomere length. An epidemiological study in China showed that prenatal exposure to certain phthalates was associated with shortened telomeres in the cord blood of newborns [34]. However, an epidemiological study in the United States revealed a positive correlation between urinary MEHP concentration and peripheral blood telomere length [35]. The discrepancy conclusions may be related to differences in exposure concentration, duration, tissue, species, etc. These findings suggest that telomeres may be one of the important targets of PAE compounds. Telomere homeostasis is essential for spermatogenesis. When the unique telomere maintenance mechanism of germ cells has been disrupted, cells will undergo devastating effects such as growth inhibition, senescence, and death. Therefore, sperm telomere length can be considered as a new biomarker of male infertility [36]. A growing number of studies have shown a tight bond between male infertility and telomere damage. Zhu et al. [1] found that the destructive effects of DEHP and MEHP-induced germ cell senescence and morphological and structural abnormalities in testicular tissue may be associated with shorter telomeres and lower TERT expression in experimental animal and cellular models. Ling et al. [37,38], based on a cohort study on the reproduction of university students, discovered that higher levels of PAHs in urine were associated with shorter sperm telomere lengths and lower sperm mitochondrial DNA copy numbers. Ling et al. [23] further demonstrated at the cellular and animal levels that benzo[a]pyrene and its metabolite BPDE induced telomeric DNA breakage, shortened telomeres, and reduced telomerase activity and TERT expression in GC-2 cells, leading to the senescence and apoptosis of spermatogonia. There is also a link between male reproductive damage caused by pollutants such as brominated flame retardants [24] and fluoride [26] and the disruption of telomere structure and function in germ cells. These findings suggest that telomere disruption is an important target for male reproductive toxicity induced by various environmental pollutants.

Damaged telomeres can be identified at the onset of meiosis, and cells with impaired telomeres will be removed from the germ cell precursor pool. During spermatogenesis, telomerase keeps high activity in germ cells to maintain the stability of sperm chromosomes and ensure that complete chromosomes can be passed on to the offspring [39]. In the present study, we found that GC-1 cells treated with different concentrations of MEHP for 48 h significantly induced cell proliferation inhibition, G_0_/G_1_ phase cell cycle arrest, and apoptosis. As telomere disorders have been proven to be associated with male infertility, in this experimental design, we also investigated the effect of MEHP on telomeres in GC-1 cells. The results showed that after the GC-1 cells were exposed to different concentrations of MEHP for 48 h, the relative telomere length of cells was significantly shortened, the telomerase activity was decreased, and the mRNA expression levels of telomere-binding protein complexes *Trf1*, *Trf2*, *Pot1*, *Rap1*, and *Tin2* were reduced. Telomerase reverse transcriptase (TERT) is a critical determinant of telomerase activity and telomere length maintenance. MEHP also decreased the expression of TERT mRNA and protein in GC-1 cells in a dose-dependent manner. Our results are similar to those of Zhu et al. These results suggest that the cytotoxic effects of MEHP-induced GC-1 cells may be associated with a decrease in TERT expression. In a sense, TERT expression may prevent MEHP-induced cell cycle arrest and apoptosis by maintaining telomere function. c-Myc is a transcription factor binding site located in the promoter region of the TERT gene, which binds to the Max protein to form a c-Myc/Max protein dimer. This dimer can conjugate to the specific site E-box on the DNA chain to acetylate histones, promote transcription, and up-regulate the expression of TERT, thus activating telomerase activity and positively regulating telomere length [15]. c-Myc can also indirectly activate the expression of TERT through the E_2_/ER pathway [16]. These hints suggest that c-Myc is a key factor in regulating TERT transcriptional expression. Our study found that MEHP reduced the mRNA and protein expression of c-Myc and Max in GC-1 cells in a dose-dependent manner, which may be associated with telomere impairing effects in male reproductive damage. This result indicates that G_0_/G_1_ phase cell cycle arrest and apoptosis in GC-1 cells induced by MEHP exposure may be related to c-Myc-mediated TERT-associated telomere damage.

To clarify the reason for the reduction in MEHP-induced c-Myc expression in GC-1 cells, we used the Cistrome DB, TRRUST, and Genecards databases to screen for c-Myc transcriptional regulators. We found a total of 37 genes in the Cistrome DB database, 74 genes in the TRRUST database, and 209 genes in the Genecards database. Visualizing the results for the unique and common sections between the groups, we found that five genes, CTCF, STAT3, ESR1, C-JUN, and FOXA1, were detected in the three databases. All five transcription factors are directly linked to male reproductive health. STAT3 is a member of the signal transduction and transcription activator protein family. The expression level of STAT3 in spermatogenic tubules is closely related to spermatogenesis [40]. JUN, as a classical transcription factor, is stably expressed in the nucleus of spermatogenic cells, widely exists in A1 spermatogonial cells, and is involved in the biological process of spermatogonial initiation and differentiation. Related research has revealed that the conditional knockout of JUN caused significant reproductive abnormality in mice such as prolonged female estrous cycles and reduced male sperm counts [41]. CTCF has a critical role in the development of male germ cells. Studies have discovered that the suppression of CTCF protein expression in spermatocytes can cause spermatogenesis disorders and infertility [42]. In conjunction with the present study, we detected that MEHP treatment inhibited the mRNA expression of *Ctcf, Stat3, Esr1, C-jun*, and *Foxa1* in GC-1 cells, while the protein expressions of CTCF, ESR1, and C-JUN were also significantly blocked. These findings indicate that the detrimental effects of MEHP on c-Myc may be associated with the impaired expression of CTCF, ESR1, and C-JUN, the upstream transcription factors of c-Myc. Given what is known about phthalates and their anti-androgenic activity, we comment on the observation of ESR1. In this study, we observed a dose-dependent decrease in mRNA and the protein expression levels of ESR1 in the GC-1 cells after 48 h of MEHP treatment, particularly in the 200 and 400 μM MEHP exposure groups. The ESR1 gene is an indispensable part of the development of the reproductive system, and its normal expression plays an important regulatory role in male reproductive function [43]. Related studies have shown that the ESR1 gene is involved in the process of spermatogenesis and sperm maturation. After the ESR1 gene is knocked out, the sperm concentration and motility in epididymis decrease significantly. Similarly, the absence of ESR1 can lead to male infertility, which may be associated with abnormalities in the spermatogenic epithelium and spermatogenesis [44]. In part, these discoveries may account for the fact that impaired ESR1 expression probably contributes directly to the damaged male reproductive system.

In summary, based on an in vitro MEHP staining model of mouse spermatogonia-derived GC-1 cells, we discovered that MEHP exposure may induce cell cycle dysregulation and apoptosis by mediating abnormal telomere structure and function in spermatogonia. The telomerase reverse transcriptase (TERT) may be involved in this damaging process to some extent. Further studies suggest that the reduced expression of TERT may be associated with a decrease in the expression of c-Myc, a transcriptional regulator that regulates TERT activity, and that there is a possible link between the blocked expression of c-Myc and diminished expression levels of CTCF, ESR1, and C-JUN, which are transcriptional regulators of c-Myc signaling. Our findings implicate that MEHP may inhibit c-Myc signaling through specific transcriptional regulators, activate TERT-mediated telomere damage, and lead to G_0_/G_1_ phase cell cycle arrest and apoptosis in GC-1 cells. However, this conclusion, only based on the present study, has limitations. There may be other causes independent of telomere damage to explain the cytotoxic damage induced by MEHP treatment. This discovery has, to some extent, enriched the molecular mechanism of MEHP-induced male reproductive damage, providing a new perspective and a unique way of thinking to study the reproductive damage in males.

## Data Availability

All of the data generated or analyzed during this study are included in this published article.

## Supplementary Materials

The following supporting information can be downloaded at: https://www.mdpi.com/article/10.3390/toxics11050448/s1, Table S1: Primers’ sequences for telomere genes and reference genes; Table S2: Primers’ sequences for various genes; Figure S1: Changes in the mRNA expression levels of *Cdk4*, *Cdk6*, *Ccnd1*, and *Rb1* in GC-1 cells after 48 h of MEHP treatment were analysed by RT-qPCR.
