# Supplementary material for: Mono-(2-ethylhexyl) Phthalate (MEHP)-Induced Telomere Structure and Function Disorder Mediates Cell Cycle Dysregulation and Apoptosis via c-Myc and Its Upstream Transcription Factors in a Mouse Spermatogonia-Derived (GC-1) Cell Line
Source: Toxics. 2023 May 10;11(5):448. doi: 10.3390/toxics11050448 (PMC10221830; doi:10.3390/toxics11050448)
Supplement: Supplementary file 1 [file toxics-11-00448-s001.zip › toxics-2307806-supplementary.pdf]

Supplemental Materials

Table S1. Primers' sequences for telomere genes and reference genes.

Gene	Forward primers (5'–3')	Reverse primers (5'–3')
Telo	CGGTTTGTTTGGGTTTGGGTTT	GGCTTGCCTTACCCTTACCCT
	GGGTTTGGGTTTGGGTT	TACCCTTACCCTTACCCT
m36B4	ACTGGTCTAGGACCCGAGAAG	TCAATGGTGCCTCTGGAGATT

Table S2. Primers' sequences for various genes.

Gene	Forward primers (5'–3')	Reverse primers (5'–3')
Cdk4	ATGGCTGCCACTCGATATGAA	TGCTCCTCCATTAGGAACTCTC
Cdk6	TCTCACAGAGTAGTGCATCGT	CGAGGTAAGGGCCATCTGAAAA
Ccnd1	GCGTACCCTGACACCAATCTC	CTCCTCTTCGCACTTCTGCTC
Rb1	TTGGAGTCCGATTGTATTACCGT	AGCACAGGCCAGTAAAGACAT
Trf1	CAGCAGTCTACAGAAACTGAACC	ACTGAAATCTGATGGAGCACG
Trf2	GGTGCTCAAGTTCTATTTCCACG	CAAGCAACGCCTGCATGAT
Pot1	AGCCTCCGTATGTTAGCAAAGG	CCTGTGGAAGCGAACAATGT
Rap1	ATGCGTGAGTACAAGCTAGTAGT	AATCTACCTCGACTTGCTTTCTG
Tin2	TGCCCTGAAGCATCACTTCC	GGCAACTAGAAAGGATTCCCC
Tpp1	GAGTCTCACTTTTGCGCTGAA	CTCCAGGGTTAGGTACTTTCCA
Tert	GCACTTTGGTTGCCCAATG	GCACGTTTCTCTCGTTGCG
c-Myc	ATGCCCCTCAACGTGAACTTC	GTCGCAGATGAAATAGGGCTG
Max	CAAGCGGGCTCACCATAATG	TGTTGCTTTGTCTAGGATTTGGG
Stat3	CACCTTGGATTGAGAGTCAAGAC	AGGAATCGGCTATATTGCTGGT
C-jun	TTCCTCCAGTCCGAGAGCG	TGAGAAGGTCCGAGTTCTTGG
Foxa1	ACATTCAAGCGCAGCTACCC	TGCTGGTTCTGGCGGTAATAG
Esr1	GACCCTTCACACCAAAGCCTC	TGTCCACGTATACCTCGCCC
Ctcf	GATCCTACCCTTCTCCAGATGAA	GTACCGTCACAGGAACAGGT
actin	CGTGCGTGACATCAAAGAGAAG	CAAGAAGGAAGGCTGGAAAAGA

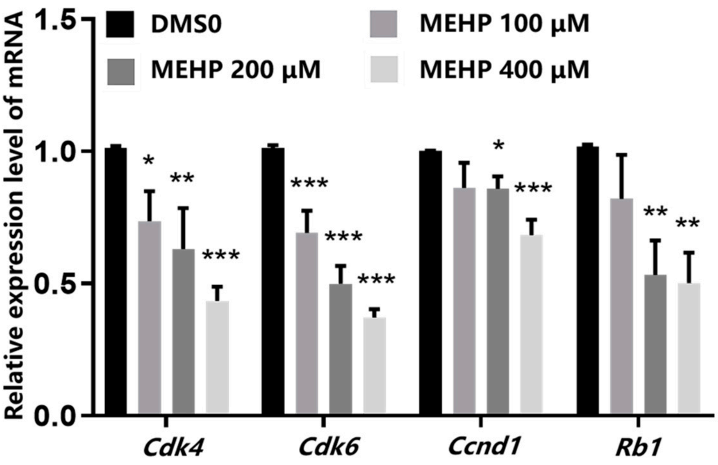

Figure S1. Changes in the mRNA expression levels of *Cdk4, Cdk6, Ccnd1,* and *Rb1* in GC-1 cells after 48 h of MEHP treatment were analysed by RT-qPCR. *Beta-actin* (*β-actin*) was used as the housekeeper gene. The results are presented as the mean ± SD, n = 3. **P* < 0.05, ***P* < 0.01, ****P* < 0.001, compared with the solvent control (DMSO) group.
